# A Novel *Lactobacillus brevis* Fermented with a Vegetable Substrate (AL0035) Counteracts TNBS-Induced Colitis by Modulating the Gut Microbiota Composition and Intestinal Barrier

**DOI:** 10.3390/nu16070937

**Published:** 2024-03-24

**Authors:** Loredana Vesci, Grazia Tundo, Sara Soldi, Serena Galletti, Daniela Stoppoloni, Roberta Bernardini, Anamaria Bianca Modolea, Laura Luberto, Emanuele Marra, Fabrizio Giorgi, Stefano Marini

**Affiliations:** 1 Corporate R&D, Alfasigma S.p.A., Via Pontina km 30.400, Pomezia, 00071 Rome, Italy; fabrizio.giorgi@alfasigma.com; 2 Department of Translational Medicine, University of Tor Vergata, Via Montpellier 1, 00133 Rome, Italy; roberta.bernardini@uniroma2.it (R.B.); modoleabianca@gmail.com (A.B.M.); stefano.marini@uniroma2.it (S.M.); 3 AAT Advanced Analytical Technologies Srl, Via P. Majavacca 12, 29017 Fiorenzuola d’Arda, Italy; sara.soldi@aat-taa.eu (S.S.); serena.galletti@aat-taa.eu (S.G.); 4 Takis Castel Romano, 00128 Rome, Italy; stoppoloni@takisbiotech.it (D.S.); luberto@takisbiotech.it (L.L.); marra@takisbiotech.it (E.M.); 5 Centro Interdipartimentale di Medicina Comparata, Tecniche Alternative ed Acquacoltura (CIMETA), University of Tor Vergata, Via Montpellier 1, 00133 Rome, Italy

**Keywords:** inflammatory bowel disease, probiotics, *Lactobacillus brevis*, gut microbiome, fermentation, intestinal barrier

## Abstract

Crohn’s and ulcerative colitis are common conditions associated with inflammatory bowel disease as well as intestinal flora and epithelial barrier dysfunction. A novel fermented *Lactobacillus brevis* (AL0035) herein assayed in a trinitro benzene sulfonic acid (TNBS)-induced colitis mice model after oral administration significantly counteracted the body weight loss and improves the disease activity index and histological injury scores. AL0035 significantly decreased the mRNA and protein expression of different pro-inflammatory cytokines (TNFalpha, IL-1beta, IL-6, IL-12, IFN-gamma) and enhanced the expression of IL-10. In addition, the probiotic promoted the expression of tight junction proteins, such as ZO-1, keeping the intestinal mucosal barrier function to attenuate colitis symptoms in mice. Markers of inflammation cascade such as myeloperoxidase (MPO) and PPAR-gamma measured in the colon were also modified by AL0035 treatment. AL0035 was also able to reduce different lymphocyte markers’ infiltration in the colon (GATA-3, T-Bet, NK1.1) and monocyte chemoattractant protein-1 (MCP-1/CCL2), a key chemokine involved in the migration and infiltration of monocytes/macrophages in the immunological surveillance of tissues and inflammation. In colonic microbiota profile analysis through 16S rRNA sequencing, AL0035 increased the microbial diversity depleted by TNBS administration and the relative abundance of the Lactobacillaceae and Lachnospiraceae families, whereas it decreased the abundance of Proteobacteria. Altogether, these data indicated that AL0035 could lower the severity of colitis induced by TNBS by regulating inflammatory cytokines, increasing the expression of tight junction proteins and modulating intestinal microbiota, thus preventing tissue damage induced by colitis.

## 1. Introduction

Crohn’s disease (CD) and ulcerative colitis (UC) are major inflammatory bowel diseases (IBDs) and affect approximately 10–20 per 100,000 people. UC is mostly circumscribed to the colonic mucosa with neutrophils predominating in the lamina propria and crypts of the colon; on the other hand, CD can involve any site of the gut, particularly the terminal ileum and colon with transmural inflammation. The etiology of IBDs is still unknown, and it is generally considered to be caused by multiple factors. Many studies suggest that the intestinal mucosal barrier and intestinal microflora play an important role in favoring the development of these diseases.

The intestinal mucosal barrier made up of epithelial cells connected by tight junctions (TJs) settles the specific movement of solutes across the epithelium and limits the entry of pathogens and detrimental substances into the submucosa, thus avoiding inflammation [1]. TJs mainly include zonula occludens (ZO) proteins connected with the transmembrane proteins claudins and occludins in order to compose a stable TJ complex [2]. A relationship between intestinal microflora dysbiosis and gut barrier function is reported. In fact, an intestinal flora disequilibrium is evident together with the impairment of the gut mucosal barrier. It causes epithelial cell death and raises gut permeability, thus promoting the pathogens and toxins’ translocation [3].

Nowadays, the main drugs used for patients with IBD are, first of all, mesalazine but also corticosteroids, immunosuppressants or biological drugs depending on the severity of the disease. The common issue of possible adverse effects induced by these drugs [4,5] prompted us to further investigate other approaches such as to improve the intestinal mucosal barrier function and to balance gut microflora for IBD treatment. Additionally, several investigations have previously revealed that probiotics are able to support good intestinal function but also to manage many inflammatory gastrointestinal disorders [6,7,8].

As a matter of fact, a novel *Lactobacillus brevis* (MZ Leibniz Institute DSMZ-Braunschweig, Germany-Collection of Microorganism and Cell Culture GmbH, DSM 33682) fermented with a vegetable substrate (not disclosed) (named AL0035) used as a technological adjuvant was identified for the first time within an in vitro screening for its anti-inflammatory activity. Therefore, the aim of this study was to investigate its anti-inflammatory effect on the intestinal barrier of experimental mice with a trinitrobenzenesulfonic acid (TNBS) that induces a severe colitis. Its possible mechanism of action was assessed by evaluating the histopathology damage, gut microbiota composition, TJ protein content, level of main inflammatory cytokines that are altered in IBD [6] and infiltration of different immune populations in the colitis mice model.

## 2. Materials and Methods

### 2.1. Bacterial Strain and Preparation

The bacterial strain *Lactobacillus* (*L.*) *brevis* (*Alfasigma Property*) was listed in the culture collection (DSMZ Leibniz Institute DSMZ-German-Collection of Microorganism and Cell Culture GmbH, DSM 33682). The inoculum of the *L. brevis* strain was grown at 37 °C for 12 h in a fermentation medium containing an aliquot of a vegetal substrate (not disclosed) that was previously sterilized. Next, the bacterial biomass was collected after centrifugation at 3000× *g* for 10 min at 4 °C and lyophilized.

### 2.2. Animals and Experimental Design

C57BL/6 male mice aged 7–8 w (ENVIGO, Udine, Italy) were used for the study. The animals were housed under standard laboratory conditions: light/dark cycles (12/12 h), ambient temperature 20 ± 2 °C, 55% relative air humidity and food (Mucedola RF18) and water ad libitum.

All experiments were approved by the Animal Welfare Body (OBPA) and carried out according to the Italian and European rules (D.L.vo 26/14; European Directive 2010/63/EU). A veterinary surgeon was present during the procedure. The animal handling, before and after the experiment, was carried out only by trained personnel.

The mice were randomly divided in different groups (*n* = 8 per each group) as follows: normal, not TNBS-treated (Control group); TNBS-induced colitis (TNBS vehicle-treated group); and TNBS-induced colitis mice treated with different types of products including the drug positive control mesalazine.

Colitis was induced in the mice as previously described [9]. Briefly, 5% (*w*/*v*) TNBS solution dissolved in 50% ethanol solution (100 mg/kg) was used to induce colitis. Excluding the Control group, the other groups were first anesthetized with ether before inserting, through their anus, a catheter of ~18 cm suitable for injecting the TNBS–ethanol solution into the colon. Then, each mouse was kept in a head-down position for 2–3 min before being put in the cage. The normal Control group was intrarectally treated with an equal volume of saline. After 3 h of TNBS-administration, all mice were orally treated for 4 days, and in the meantime, they were routinely controlled for their body weight and diarrhea. All mice were sacrificed by cervical dislocation for the sample collection at the end of day 4. The evaluation of the disease activity was determined using an established scoring system [9,10]. The disease activity index (DAI) was calculated by assigning well-established and validated scores for parameters that are somewhat analogous to the clinical presentation of human IBD [11] (see Table 1). The DAI (see Table 1) was calculated as the total of these scores: the sum of weight loss, diarrhea and bleeding, resulting in the total DAI score ranging from 0 (unaffected) to 12 (severe colitis).

### 2.3. Sample Collection and Evaluation of the Macroscopic Score

The mice were sacrificed by cervical dislocation. After the abdomen opening, the colon rectum was removed, placed on an ice plate, and measured. The tissue was longitudinally opened to determine the gross macroscopic damage rate. The macroscopic characteristics of the disease were evaluated by a previously established scoring system (see Table 2) [9,12].

### 2.4. Colonic Histopathology

Colon tissues were collected and fixed into 4% buffered paraformaldehyde solution overnight at 4 °C. The following day, the tissues were rinsed with distilled water and incubated for 10 min at room temperature (three times). Then, distilled water was replaced with increasing concentrations of ethanol solution: 50% for 15 min, 70% for 15 min and 80% for 15 min for the dehydration process. The tissues were cleaned twice with xylene before being embedded in paraffin. The paraffin sections were cut into slices of 3 μm and stained with H&E staining solution. Finally, the stained sections were observed and photographed under a light microscope. The colon mucosa damage index (CMDI) score was assessed in a blinded fashion, as described in Table 3 [13,14]. The CMDI was calculated by combining the histological scores for tissue damage and the infiltration of inflammatory cells, ranging from 0 (no changes) to 6 (extensive cell infiltration and tissue damage).

### 2.5. Myeloperoxidase (MPO) Activity

Colonic mucosal scrapings from 1 cm of the colon were suspended in potassium phosphate buffer (pH 6.0) with hexadecyl trimethylammonium bromide buffer supplemented with a cocktail of protease inhibitors. The samples were then homogenized on ice and sonicated. Then, the suspensions were centrifuged at 10,000× *g* for 10 min at 4 °C, and the supernatants were diluted in potassium phosphate buffer (pH 6.0) containing 0.167 mg O-dianisidine dihydrochloride and 0.0005% (*v*/*v*) H_2_O_2_. Changes in absorbance at 450 nm were recorded with a spectrophotometer [10,15].

### 2.6. Relative Quantitative Real-Time PCR

The colonic cells were treated with Trizol (Life-technologies, Segrate, Milano, Italy) to isolate the RNA. First-strand cDNAs were synthesized from 1 µg of total RNA in a 20 µL reaction with reverse transcriptase (BioLine, Wildong Road, Memphis, TN, USA). Real-time PCR was performed using SYBR green Master Mix (Biorad, Segrate, Milano, Italy). GAPDH was used as the internal control. All primers are reported in Table 4. The relative transcription mRNA level was calculated.

### 2.7. Western Blot Analysis

The mucosa was scraped from the colon and stored at −80 °C until use. It was then immersed in urea extraction buffer (6 M Urea, 0.1% Triton X-100, 10 mM Tris, pH 8.0, 1 mM DTT, 5 mM MgCl_2_, 5 mM EGTA, 150 mM NaCl), supplemented with PMSF and an inhibitor cocktail of proteases (Sigma Aldrich, Milano, Italy) to prevent protein degradation. For membrane-bound proteins, the samples were sonicated for 25 s. The protein concentration was determined by a Bradford assay, and the samples were run on an 8% acrylamide gel for ZO-1 and NK1.1; a 10% acrylamide gel for Gata-3, IL-1β and IL-12; and T-bet and 15% acrylamide gel for INF-γ, TNF-α, MCP1-1¸ IL-10 and IL-6, under denaturing and reducing conditions. The proteins were then transferred to nitrocellulose filters and unsaturated binding sites blocked with 5% non-fat milk for 1 h. The filters were then incubated overnight at 4 °C with the antibody specific for the examined protein and with a species-specific HRP-conjugated secondary antibody. Actin and/or GAPDH were used as the internal control. Immunoreactive bands were detected using a chemiluminescence kit according to the manufacturer’s instructions (Life technologies), and the image was acquired through a C280 Azure Biosystem documentation system. The densitometric analysis of the bands was performed by ImageJ software 2.0.

### 2.8. Stool DNA Extraction and Microbiota Analysis

Mice feces were collected and stored at −80 °C after snap freezing in liquid nitrogen. The bacterial DNA of each sample was extracted using the FastDNA SPIN Kit for soil and the FastPrep Instrument (MP Biomedicals, Santa Ana, CA, USA), according to the manufacturer’s protocols.

The V4-5 hypervariable regions of the bacterial 16S rRNA gene were amplified and sequenced by the Integrated Microbiome Resource Institute (Dalhousie University, Halifax, NS, Canada) to obtain the microbial composition of the analyzed samples. Amplicon libraries were generated with primers based on the 515FB (5′-GTGYCAGCMGCCGCGGTAA-3′)/926R (5′-CCGYCAATTYMTTTRAGTTT-3′), as suggested previously [16]. The sequencing instrumentation, methodology and chemistry were based on the Illumina MiSeq instrument using the 2 × 300 bp paired-end v3 chemistry, as detailed by [17].

All the data were based on sequenced reads and operational taxonomic units.

### 2.9. Statistical Analysis

**Figure 1:**
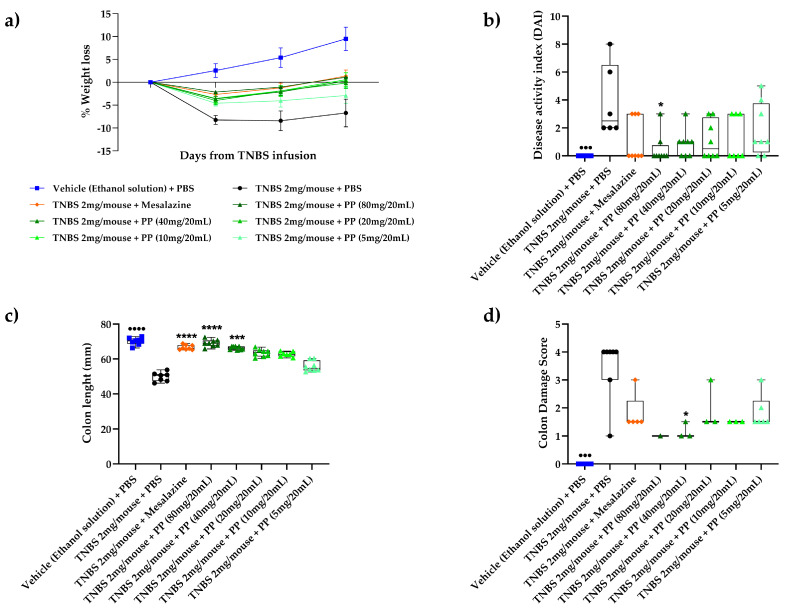
(**a**) Body weight change in TNBS-induced colitis treated with AL0035. For all three time points, a *t*-test for Vehicles vs. TNBS was applied, highlighting significative differences with a *p*-value < 0.001 (^●●●^); (**b**) DAI score; (**c**) Colon length; (**d**) Colon Damage score. For figures (**b**–**d**), a *t*-test for Vehicles vs. TNBS was first applied with a *p*-value < 0.001 (^●●●^) and *p* < 0.0001 (^●●●●^). A One-Way ANOVA (or equivalent) was applied to compare all other groups to TNBS with a *p*-value < 0.05 (*), *p* < 0.001 (***) and *p* < 0.0001 (****).

GraphPad Prism version 9.5 (GraphPad, Inc., La Jolla, CA, USA) was used for statistical analyses. The results were indicated as the mean ± standard error (SE). In detail, for BWL (Figure 1a) at all three time points, a *t*-test for Vehicles vs. TNBS was applied to highlight significative differences with a *p*-value < 0.001 (^●●●^). For the other parameters represented in Figure 1, including DAI and colon length/damage scores (Figure 1b–d), a Student’s *t*-test (parametric or non-parametric according to normal distribution of data) was applied, comparing the Vehicle vs. TNBS (^●●^ *p* < 0.01; ^●●●^ *p* < 0.001 and ^●●●●^ *p* < 0.0001), followed by One-Way ANOVA (or the corresponding non-parametric Kruskal–Wallis) with Dunnett’s multiple comparison test (or Dunn’s multiple comparisons test) for TNBS vs. different dosages of AL0035 (* *p* < 0.05; ** *p* < 0.01; *** *p* < 0.001 and **** *p* < 0.0001). For all the other figures, the One-Way ANOVA was applied between TNBS vs. AL0035 and mesalazine treatments (* *p* < 0.05; ** *p* < 0.01; *** *p* < 0.001 and **** *p* < 0.0001).

## 3. Results

### 3.1. Lactobacillus brevis (AL0035) Alleviates Clinical Symptoms of TNBS-Induced Colitis in Mice

The TNBS-vehicle-treated group generated weight loss, diarrhea and rectal bleeding like the clinical symptoms of colitis shown in Figure 1a, differently from the normal Control group. In fact, a significant decrease in body weight and a rise in the DAI score (*p* < 0.001) compared to those of the Control group were found. Both the AL0035 and MS groups were shown to counteract the body weight loss and DAI scores observed in the TNBS-vehicle-treated group (Figure 1b). These results clearly indicate that AL0035 alleviated TNBS-induced colitis signs, also reinforced by the analysis of the colon length and colon damage (Figure 1c,d).

### 3.2. AL0035 Ameliorates Intestinal Injury in TNBS-Induced Colitis Mice

**Figure 2:**
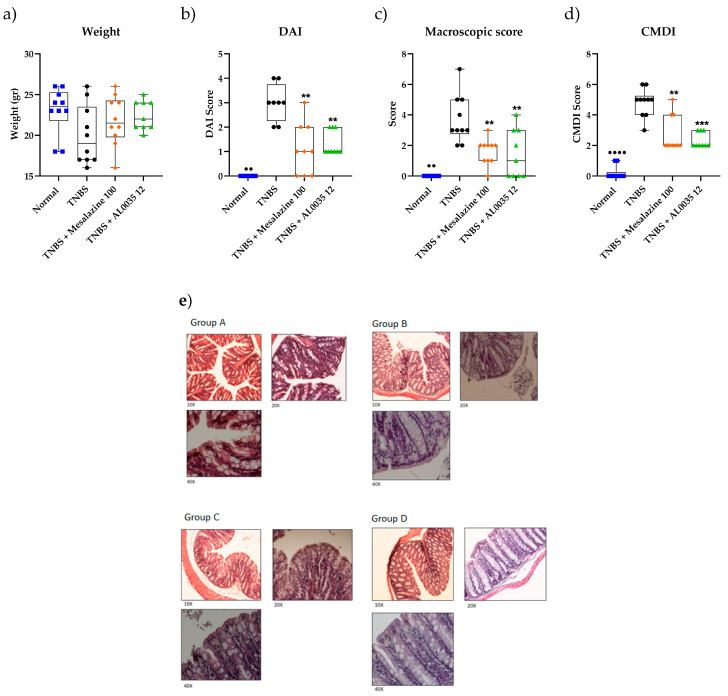
(**a**) Body weight change for TNBS-induced colitis treated with AL0035; (**b**) DAI score; (**c**) Colon length; (**d**) Colon Damage score. A *t*-test for Vehicles vs. TNBS was first applied with a *p*-value < 0.01 (^●●^) and *p* < 0.001 (^●●●●^). One-Way ANOVA (or equivalent) was applied to compare all other groups to TNBS with a *p*-value < 0.01 (**) and *p* < 0.0001 (***); (**e**) Histopathological representative images of the colon (group A, normal; group B, TNBS; group C, mesalazine; group D, AL0035).

A single dose of 12 mg/kg was selected after a curve dose-response in order to obtain a dose able to decrease the colon damage score by 50%. This amount of AL0035 was administered according to the schedule mentioned above. The treatment showed comparable activity to that of mesalazine (MS), as a result of the DAI score, body weight change and colon length and score (Figure 2a–d). Histopathological examination showed the disappearance of many epithelial cells in the tissue mucosa of intestinal glands, the infiltration of inflammatory cells, epithelial necrosis and exfoliated epithelial cells in the colon tissue of mice injected with TNBS (with black arrows) (group B). The histologic samples of the AL0035 (12 mg or 7.8 × 10^9^ CFU kg^−1^) (group D) or MS groups (100 mg/kg, po) (group C) indicated progressive restoration and a reduction in edema and necrosis as compared to those in the TNBS group. No lesions were observed in the colon tissue of the Control mice (group A), as exemplified in the histopathological samples reported in Figure 2e. Although the colon tissue of the AL0035-group mice showed some inflammatory lesions, the severity of inflammation was less than that in the TNBS mice (*p* < 0.001; Figure 2).

### 3.3. AL0035 Affects Inflammatory Cytokines, the PPAR-γ level and MPO Activity in the Colon

**Figure 3:**
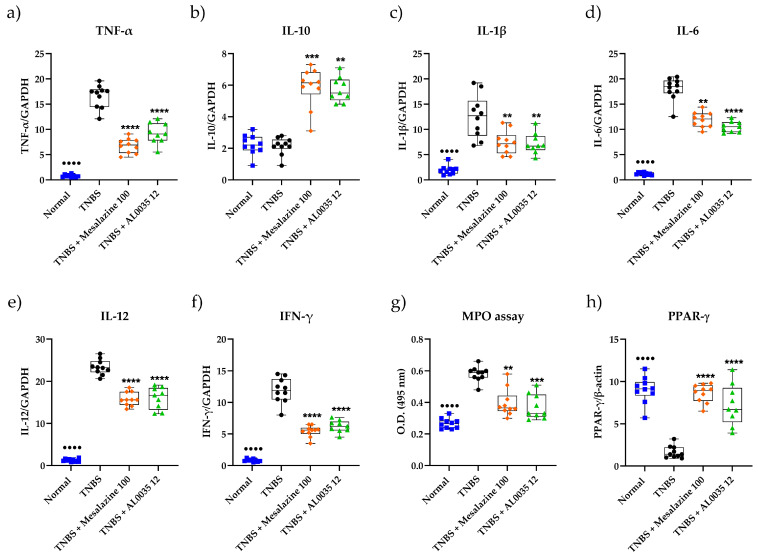
Pro-inflammatory cytokines (**a**) TNF-α; (**c**) IL-1β; (**d**) IL-6; (**e**) IL-12; (**f**) IFN-γ; anti-inflammatory cytokine (**b**) IL-10; (**g**) MPO levels and (**h**) PPAR-gamma measured in colons of TNBS-induced colitis-treated with AL0035. A *t*-test for Vehicles vs. TNBS was first applied with a *p*-value < 0.0001 (^●●●●^). A One-Way ANOVA (or equivalent) was applied to compare all other groups to TNBS with a *p*-value < 0.01 (**), *p* < 0.001 (***) and *p* < 0.0001 (****).

The levels of the inflammatory cytokines TNF-α, IL-1β, IL-6, IL-12 and IFN-γ in the colon tissue were significantly enhanced in the TNBS group with respect to those in the Control group (*p* < 0.0001), whereas treatment with mesalazine or AL0035 significantly restrained the pro-inflammatory cytokine levels (*p* < 0.0001) (Figure 3a,c–f). In the TNBS-induced colitis group, the anti-inflammatory cytokine IL-10 levels in colonic tissue were significantly suppressed (*p* < 0.01 or *p* < 0.001) (Figure 3b), suggesting that both AL0035 and mesalazine treatments revealed to slow down this process. Also, TNBS-induced colitis significantly increased the MPO activity in the colon compared to that measured in the Control mice (*p* < 0.0001), while the AL0035 and MS groups were shown to have significantly decreased MPO accumulation in colonic tissue compared to the TNBS-vehicle-treated group (*p* < 0.01 and *p* < 0.001; Figure 3g). These results indicated that AL0035 and mesalazine have a significant effectiveness in easing the severity of colitis. In addition, PPAR-γ resulted in a downregulated colon in the TNBS-induced colitis group, whereas AL0035 and mesalazine counteracted this downregulation (*p* < 0.0001) (see Figure 3h).

### 3.4. AL0035 Affects the Expression of Inflammatory Cytokines in TNBS-Treated Mice

**Figure 4:**
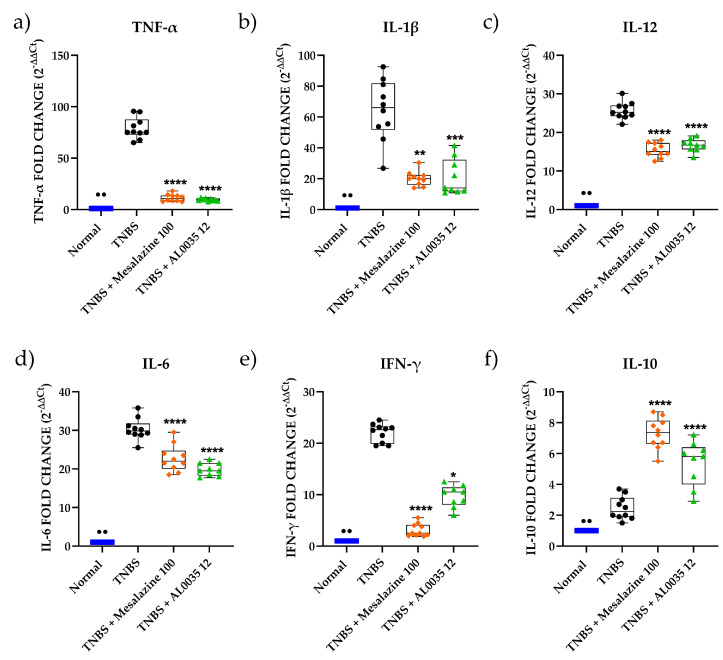
mRNA of pro-inflammatory cytokines (**a**) IL-1β; (**b**) TNF-α; (**c**) IL-12; (**d**) IL-6; (**e**) IFN-γ and mRNA of anti-inflammatory cytokine (**f**) Il-10 in colons of TNBS-induced colitis-treated with AL0035. A *t*-test for Vehicles vs. TNBS was first applied with a *p*-value < 0.01 (^●●^). A One-Way ANOVA (or equivalent) was applied to compare all other groups to TNBS, with a *p*-value < 0.05 (*), *p* < 0.01 (**), *p* < 0.001 (***) and *p* < 0.0001 (****).

In the TNBS group, compared with the Control group, the pro-inflammatory cytokines’ (IL-1β, TNF-α, IL-12, IL-6, IFN-γ) mRNA levels, detected by quantitative real-time PCR (RT-PCR), markedly increased (*p* < 0.01) (Figure 4a–e), whereas the anti-inflammatory cytokine IL-10 mRNA level (*p* < 0.01) significantly decreased (Figure 4f).

With respect to the TNBS group levels, treatment with AL0035 or mesalazine significantly inhibited pro-inflammatory cytokines (*p* < 0.05-*p* < 0.0001) levels and favoured the expression of IL-10 (*p* < 0.0001) (Figure 4).

### 3.5. AL0035 Ameliorates the TNBS-Induced Tight Junction Protein Decrease

**Figure 5:**
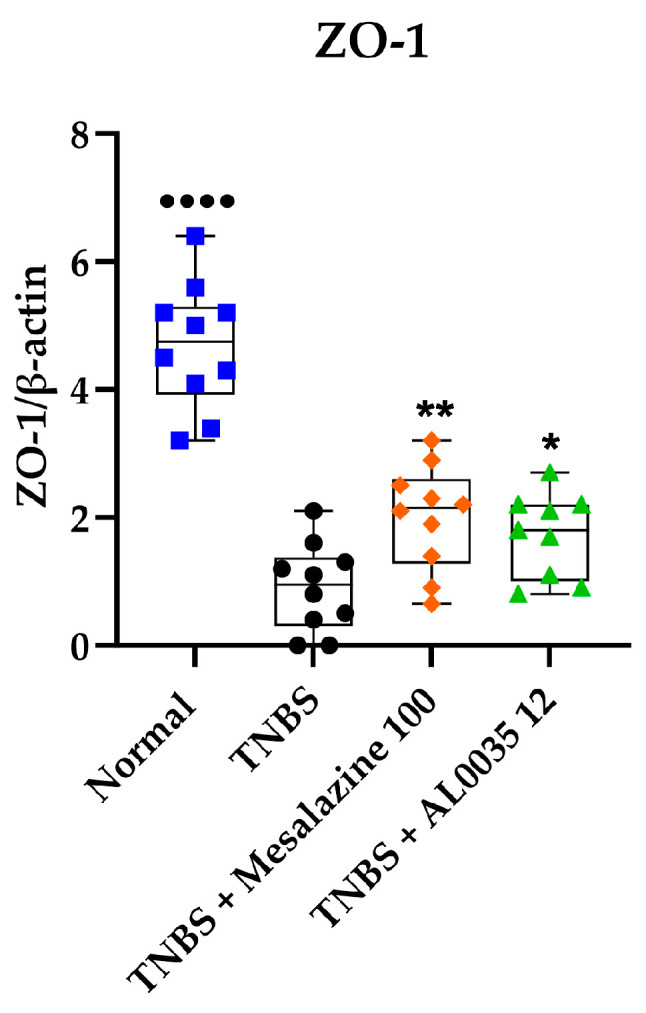
Zonula occludens 1 (ZO-1) was measured by western blotting in colons of TNBS-induced colitis-treated with AL0035. A *t*-test for Vehicles vs. TNBS was first applied with a *p*-value < 0.0001 (^●●●●^). A One-Way ANOVA (or equivalent) was applied to compare all other groups to TNBS with a *p*-value < 0.05 (*) and *p* < 0.01 (**).

To quantify the expression of TJ proteins in different groups, the western blotting analysis was carried out. With respect to the Control group, the TNBS-treated mice showed a lower expression of ZO-1, whereas compared to the TNBS group, the AL0035- or mesalazine-treated group presented a significantly increased expression of ZO-1 (*p* < 0.05) or (*p* < 0.01) (Figure 5).

### 3.6. AL0035 Treatment Reduces Lymphocyte Markers Infiltration in the Colons of TNBS-Induced Colitis Mice

**Figure 6:**
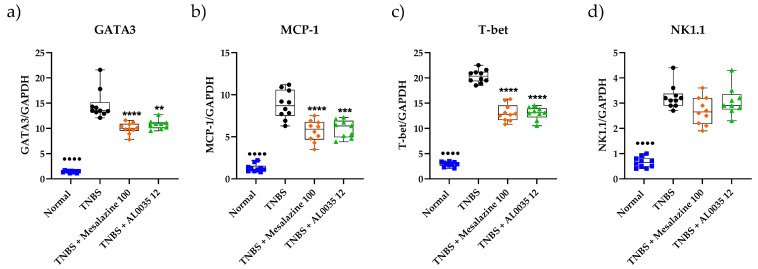
The relative expression of immune cells (**a**) GATA-3; (**b**) MCP-1; (**c**) T-Bet; (**d**) NK1.1 was measured by western blotting in colons of TNBS-induced colitis treated with AL0035. A *t*-test for Vehicles vs. TNBS was first applied with a *p*-value < 0.0001 (^●●●●^). A One-Way ANOVA (or equivalent) was applied to compare all other groups to TNBS with a *p*-value < 0.01 (**), *p* < 0.001 (***) and *p* < 0.0001 (****).

**Figure 7:**
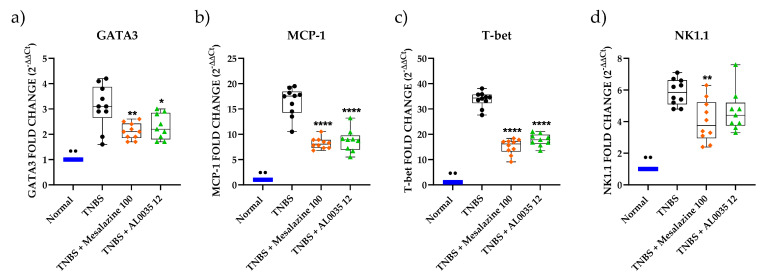
mRNA of immune cells (**a**) GATA-3; (**b**) MCP-1; (**c**) T-Bet; (**d**) NK1.1 measured by real time PCR in colons of TNBS-induced colitis treated with AL0035. A *t*-test for Vehicles vs. TNBS was first applied with a *p*-value < 0.01 (^●●^). A One-Way ANOVA (or equivalent) was applied to compare all other groups to TNBS with a *p*-value < 0.05 (*), *p* < 0.01 (**) and *p* < 0.0001 (****).

The western blot analysis of different lymphocytes populations (GATA-3, MCP-1, T-Bet, NK1.1) revealed a significant decrease induced by AL0035 or mesalazine (Figure 6a–d). Moreover, a reduction in the level of Monocyte chemoattractant protein-1 (MCP-1/CCL2) was also found after AL0035 or mesalazine treatment. Analogue results were observed by the qPCR evaluation of the same immune cells population (Figure 7a–d).

### 3.7. AL0035 Treatment Shows Eubiotic Properties on the Gut Microbiota of TNBS-Induced Colitis Mice

**Figure 8:**
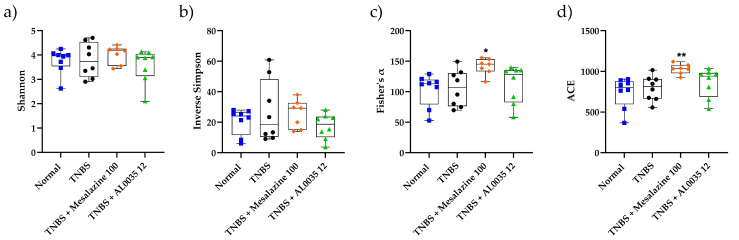
Alfa-diversity indices (**a**) Shannon; (**b**) Inverse Simpson; (**c**) Fisher; (**d**) ACE were measured in the stools of TNBS-induced colitis of mice treated with AL0035. A *t*-test for Vehicles vs. TNBS was first applied without significant differences between the two groups. A One-Way ANOVA (or equivalent) was applied to compare all other groups to TNBS with a *p*-value < 0.05 (*) and *p* < 0.01 (**).

The V3-V4 region of the 16S rRNA gene amplified from the colonic DNA-extracted samples was analyzed. The microbiota diversity was determined according to the Fisher’s Shannon and Simpson indices by using the observed bacterial species. Among the four groups, no statistical difference was observed for the Shannon, and Simpson indices, whereas in the AL0035 and MS groups, with respect to the TNBS group, the Fisher’s index turned out to be higher (Figure 8a–d). The observed species in the TNBS-treated groups were similar to those in the Control group (*p* > 0.05), whereas AL0035 tended to significantly increase the abundance of the observed species. These results suggest that AL0035 treatment increased the diversity of the gut bacterial microflora.

### 3.8. Effectiveness of AL0035 Treatment Reagrding Microbial Abundance at the Phylum and Family Levels in TNBS-Induced Colitis

**Figure 9:**
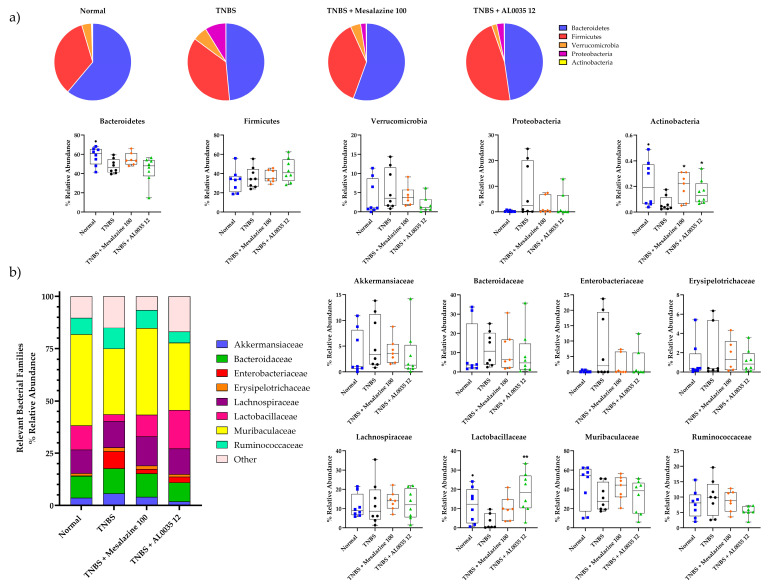
Beta-diversity as dominant phyla Firmicutes, Bacteroidetes, Proteobacteria, Verrucomicrobia and Actinobacteria and families were measured in stools of TNBS-induced colitis-treated mice. (**a**) Dominant phyla; (**b**) Dominant families. A *t*-test for Vehicles vs. TNBS was first applied: *p*-value < 0.05 (^●^). A One-Way ANOVA (or equivalent) was applied to compare all other groups to TNBS with a *p*-value < 0.05 (*) and *p* < 0.01 (**).

The microbial taxonomic composition and the comparisons among the experimental groups regarding the phylum are shown in Figure 9a. The colonic-dominant bacterial phyla observed were Firmicutes, Bacteroidetes, Proteobacteria, Verrucomicrobia and Actinobacteria.

In the detailed taxonomic analysis, the TNBS group presented a decrease in the relative abundance of Bacteroidetes and Actinobacteria and a rise in the relative abundance of Proteobacteria with respect to the normal group. In contrast, the abundance of the phylum Firmicutes was increased in the AL0035 and mesalazine groups, whereas Proteobacteria was found to be downregulated (Figure 9a).

At the family level, the relative abundance of some of the core microbiota, including *Ruminococcaceae*, *Enterobacteriaceae*, *Erysipelotrichaceae*, *Lactobacillaceae* and *Muribaculaceae*, was different in the TNBS group as compared with the relative abundance in the Control group. TNBS strongly raised the relative abundance of pro-inflammatory bacteria belonging to Proteobacteria, particularly *Enterobacteriaceae*, which were, to the contrary, reduced by the intervention with mesalazine and AL0035 in a similar manner. However, AL0035 treatment not only reduced the proportion of pro-inflammatory bacteria but also significantly increased *Lactobacillaceae*, *Muribaculaceae* and *Lachnospiraceae*, which are known to be protective towards colonic mucosa (Figure 9b).

## 4. Discussion

The 2,4,6-trinitrobenzenesulfonic acid (TNBS) colitis induced in mice is one of the main models in the experimental studies of inflammatory bowel disease (IBD) because the inflammation induced by TNBS mimics several peculiarities of Chron’s disease. It is characterized by colon inflammation accompanied by diarrhea, hemoccult, body weight loss, shortening of the colorectum and intestinal mucosal barrier impairment [18]. In the present investigation, TNBS induced damage to the intestinal epithelium as transmural colitis and a strong reduction in the body weight of animals. The effectiveness of AL0035 treatment has been highlighted through the measurement of several biomarkers. The probiotic exerted a protective effect on mice with TNBS-induced colonic injury. Importantly, the activity of MPO, which is a well-known marker of tissue damage and neutrophil infiltration involved in mucosal disruption and ulceration [19], with a close relationship with UC [20], decreased in the colon tissues of mice in the AL0035 group, suggesting that AL0035 treatment displays an anti-inflammatory effect.

It was known that the inflammatory cytokines level measured in colon tissue is linked to the progression of IBD and the efficacy of therapy [21]. Thus, we evaluated the expression levels of pro-inflammatory genes such as IL-1β and TNF-α and of the anti-inflammatory gene IL-10 in colon tissues. IL-1β and TNF-α are recognized to affect the inflammation rate in IBD patients; in fact, their increment positively correlates with the severity of IBD [22,23,24]. The treatment of TNBS-induced acute colitis with mesalazine or AL0035 reduced the expression levels of pro-inflammatory genes in mice. On the other hand, IL-10 has been demonstrated to play a crucial role in the pathogenesis of IBD. Some microbiome-based therapies such as fecal microbial transplantation and probiotics have been indicated to modulate IL-10 immune pathways and inhibit experimental colitis [4,25]. These observations point out that the regulation of IL-10 signaling should be pursued for the treatment of colitis. In the current investigation, the treatment of TNBS-induced colitis with both mesalazine and AL0035 significantly increased the expression of IL-10, which is reported to be a critical point for the decrease in gut inflammation.

In addition to the regulation of inflammatory mediators, the disruption of epithelial barrier function is another well-known pathophysiologic hallmark of IBD [22,26]. The increased secretion of pro-inflammatory cytokines leads to the damage of the intestinal mucosa and the consequent loss of intestinal permeability to harmful microorganisms, thus aggravating intestinal inflammation (and forming a vicious cycle) [23]. Particularly, TNF-α overexpression can enhance local or systemic inflammation, leading to the impairment of TJ and the gut mucosal barrier [3]. As a key element of the intestinal mucosal barrier, TJ proteins close the gap between the adjoined gut epithelial cells by inducing a protection from the confining microorganisms and antigens in the intestinal lumen. In another study, a reduced TJ-related proteins expression was found in the intestinal mucosa of IBD patients, including the intracytoplasmic protein ZO-1 and the transmembrane proteins claudin-1 and occludin [27]. In this study, we observed a significant upregulation of ZO-1 in the colon tissues of TNBS-induced colitis mice after the administration of either AL0035 or mesalazine with respect to the low levels detected in the damaged colon by TNBS.

It was previously reported that probiotics improve the intestinal barrier function, thus modulating one of the most notable factors necessary to ease the IBD symptoms [8,28]. Accordingly, the probiotics investigated in this study might be able to prevent and treat IBD by enhancing the function of the intestinal barrier so much that it deserves to be tested in clinical trials. It is to be noted that when the intestinal mucosal barrier is compromised, microorganisms and harmful substances can pass from the intestinal cavity, producing changes in the gut environment. Consequently, the dynamic balance and the gut microbiota peculiarities are also changed.

Thereafter, the gut microbiota profiling in mice of different treatment groups was compared. The Shannon and Simpson indices did not reveal differences in the richness of species among the groups. However, the results about the observed species denoted a significant difference in the TNBS group with respect to the Control group. After the AL0035 and mesalazine treatment, the diversity was improved, and the community composition changed.

On the whole, the composition of gut microbiota was closely related to IBD pathogenesis. At the phylum level, the administration of AL0035 decreased the percentages of Proteobacteria, documented as responsible for the production of enterotoxins that often cause gastroenteritis or anaphylaxis [29,30]. At the family level, the relative abundance of *Lachnospiraceae* and *Muribaculaceae*, depleted by TNBS, was recovered and further enriched in mice treated with AL0035, with an improvement correlated with the ability to produce SCFA and keep eubiotic conditions, respectively recognized in these families [31,32,33]. Significant differences were found in the *Lactobacillaceae* family, which were strongly reduced by the application of TNBS but also significantly increased by the administration of AL0035. The capacity of *Lactobacillaceae* to improve inflammatory bowel disease (IBD) and regulate the immune system is especially remarkable and well known and is due to several factors such as the production of protective molecules or downregulation in the production and release of pro-inflammatory cytokines (IL-6, IL-1β, IL-2 and TNF-α) [33].

This study shows that AL0035 can promote the proliferation of beneficial bacteria in the intestinal tract and inhibit the proliferation of potentially harmful populations. This effect on the composition of intestinal microbiota results in the improvement of IBD.

All the above results would support the anti–inflammatory effect obtained in AL0035-treated groups by means of the reduction in pro–inflammatory bacteria and the maintenance of intestinal barrier integrity and functions by the modulation of tight junction proteins (ZO-1) in the colonic tissue and the regulation of anti/pro-inflammatory mediators, displaying the same ability of mesalazine in relieving TNBS-induced colitis symptoms.

Considering this, and according to the rising literature about the administration of probiotics as a promising strategy in the treatment of different human diseases, AL0035 could be an optimal candidate for IBD treatment.

The significant anti-inflammatory and immunomodulant efficacy of AL0035, comparable to that of the drug mesalazine in the TNBS-colitis murine model, clearly represents a strength point of the novel probiotic, leading to its therapeutic use. On the other hand, it should be taken into account that animal IBD models do not completely replicate the human immunological profile, and for this reason, the pharmacological activity of probiotics should be tested in different models, also in association with standard therapy, in order to better support the design of an appropriate clinical study.

## 5. Conclusions

The novel probiotic AL0035 *Lactobacillus Brevis* fermented with a vegetal substrate as an adjuvant technology significantly counteracted the inflammation in the TNBS-induced severe colitis in a fashion comparable to that of the reference compound mesalazine, which is the standard of care of medium–moderate colitis in humans. AL0035 was also able to alleviate gut dysbiosis through the suppression of the Proteobacteria population and gut microbiota endotoxins release. The overall data reported here highlighted the therapeutic value of this novel probiotic, providing a perspective on its use in patients with IBD

## Figures and Tables

**Table 1:** Disease activity index (DAI).

DAI Index	Weight Loss (%)	Stool Consistency	Occult/Gross Bleeding
0	None	Normal	No bleeding
1	1–5		
2	5–10	Loose stools	Hemoccult-positive (slight bleeding)
3	10–15		
4	>15	Diarrhea	Gross bleeding

**Table 2:** Macroscopic score.

Colon Damage	Score
No damage	0
Hyperemia without ulcers	1
Hyperemia and wall thickening without ulcers	2
One ulceration site without wall thickening	3
Two or more ulceration sites	4
0.5 cm extension of inflammation or major damage	5
1 cm extension of inflammation or severe damage	6–10 (the score was increased by 1 for every 0.5 cm of damage up to a maximal score of 10)

**Table 3:** Colon mucosa damage index (CMDI).

**Infiltration of Inflammatory Cells**	**Score**
Presence of occasional inflammatory cells in the lamina propria	0
Increased numbers of inflammatory cells in the lamina propria	1
Confluence of inflammatory cells, extending into the submucosa	2
Transmural extension of the infiltrate	3
**Tissue damage**	**Score**
No mucosal damage	0
Discrete lymphoepithelial lesions	1
Surface mucosal erosion or focal ulceration	2
Extensive mucosal damage and extension into deeper structures of the bowel wall	3

**Table 4:** Primers list of the qPCR experiments.

Gene Name	Sequence
FoxP3	AGACCCCTGTGCTCCAAGTG CAGACTCCATTTGCCAGCAG
GATA3	GAACCGCCCCTTATCAAG CAGGATGTCCCTGCTCTCCTT
NK1.1	TCCCTTCTCACCACCAGTTA CAGTCTTGTGGGCACTCTAA
T-bet	AATCGACAACAACCCCTTTG AACTGTGTTCCCGAGGTGTC
IL-10	ATTTGAATTCCCTGGGTGAGAAG CACAGGGGAGAAATCGATGAC
IL-6	AGGATACCACTCCCAACAGACCT CAAGTGCATCATCGTTGTTCATAC
MCP-1	TGATCCCAATGAGTAGGCTGGAG ATGTCTGGACCCATTCCTTCTTG
INF-γ	CAATGAACGCTACACACTGC CCACATCTATGCCACTTGAG
TNF-α	CCCCAAAGGGATGAGAAGTTC TGAGGGTCTGGGCCATAGAA
IL-1β	TCAGGCAGGCAGTATCACTC CTAATGGGAACGTCACACC
IL-12	CAGAAGCTAACCATCTCCTGGTTTG TCCGGAGTAATTTGGTGCTTCACAC
GAPDH	AACTTTGGCATTGTGGAAGG CACATTGGGGGTAGGAACAC

## Data Availability

Other datasets analyzed during the study are available from the corresponding authors on reasonable request. The data are publicly available.
